# Asylums in India

**Published:** 1893-02-11

**Authors:** 


### ASYLUMS IN INDIA.

### II.—THE MADRAS PRESIDENCY.

In this Presidency there are three lunatic asylums, with an aggregate capacity for 729 patients, Madras having accommodation for 529, Calicut for 145, and Waltair for 55; only at Calicut does there appear to have been actual over-crowding, the maximum number of inmates on any one night having been three in excess of the proper number. The total insane population of these institutions during 1891 was 810, of whom 597 remained from the previous year, while 213 were admitted during 1891; of these 104 were discharged and 114 died, leaving 592 under treatment at the close of the year. The death-rate amounted to 18·57 per cent., an enormously high percentage, for which the Government considers no adequate explanation is forthcoming. At Calicut the death-rate declined from 20·13 in 1890 to 9·21 in 1891, and at Waltair it also declined from 11·29 to 7·6, so that the Madras Asylum, which had a death-rate of 18·7 in 1890, and of 23·09 in 1891 is alone responsible for the total increase in the death-rate from 18·38 to 18·57. The Government order, with which the report under review concludes, contains an expression of opinion that "the administration of the Madras Asylum demands the closest supervision by the surgeon-general"; and while we have no wish to express a judgment formed solely upon documentary evidence, and without having had an opportunity of making a personal inspection of the institution, we must say that we are glad to note the critical tone of the order, and that we shall await with considerable interest the next report upon the working of this particular asylum.

The percentage of cures to daily average strength in all three asylums was 9·94. The total number of criminal insane under treatment during the year was 171, with a daily average strength of 144·77 and 9 deaths, as compared with a total population of 159 in 1890, with a daily average strength of 138·88 and 15 deaths.

The total sum expended during 1891 in maintaining these three asylums was Rs. 1,42,397 11a., against Rs. 1,18,236 14a. 2p. the previous year, the excess being wholly due to an increase in the Public Works charges, which amounted to Rs. 49,035 15a. 3p., as compared with Rs. 21,283 7a. 2p. in 1890. Altogether, therefore, the asylums were worked more economically in 1891. The cost per head of average strength was Rs. 150 1a. at the Madras Asylum—a sum to which the Government raises a strong objection—Rs. 97 5a. 7p. at Waltair, and Rs. 94 13a. 1p. at Calicut.

This report is not without interest, as a piece of special pleading, but it is with somewhat mixed feelings that we lay it aside. We shall hope for better things this year.

